# User evaluation of registry‐supported recruitment: The Collaborative Approach for Asian Americans, Native Hawaiians, and Pacific Islanders (AANHPI) Research and Education (CARE)

**DOI:** 10.1002/alz.091838

**Published:** 2025-01-09

**Authors:** Marian Tzuang, Joshua D Grill, Janice Y. Tsoh, Daren Huang, Bora Nam, Dolores Gallagher‐Thompson, Ladson Hinton, Oanh L. Meyer, Van Ta Park

**Affiliations:** ^1^ University of California San Francisco School of Nursing, San Francisco, CA USA; ^2^ Institute for Memory Impairments and Neurological Disorders, University of California, Irvine, Irvine, CA USA; ^3^ Asian American Research Center on Health (ARCH), University of California San Francisco, San Francisco, CA USA; ^4^ University of California San Francisco School of Medicine, San Francisco, CA USA; ^5^ Stanford University School of Medicine, Stanford, CA USA; ^6^ University of California, Davis School of Medicine, Sacramento, CA USA

## Abstract

**Background:**

The Collaborative Approach for Asian Americans, Native Hawaiians, and Pacific Islanders (AANHPI) Research and Education (CARE) is a recruitment registry that has enrolled over 10,000 AANHPI participants who expressed willingness to participate in Alzheimer’s disease and related dementias (ADRD), aging, caregiving, and other health research. We report survey results from 24 of the 28 study principal investigators (“users”) who utilized CARE between January 2021 and October 2023 to support their study recruitment.

**Method:**

Users answered five questions on a 4‐point Likert scale (0 = Strongly disagree to 3 = Strongly agree) related to (1) user experience, (2) usefulness in accelerating recruitment, (3) improving AANHPI representation, (4) whether they would use the registry again, and (5) whether they would recommend the registry to others. The survey also included open‐ended questions for suggestions to improve the registry and collect information on products (e.g., publications) for each of the CARE‐supported studies.

**Result:**

Among 24 CARE‐supported studies with available user survey data, 14 (58%) were related to ADRD or dementia caregiving, 10 (42%) recruited specific AANHPI sub‐groups (e.g., Chinese, Korean), and 10 (42%) were funded by the National Institutes of Health. Half of users learned about CARE through word of mouth. All users agreed or strongly agreed they would recommend the registry to others and that the referral request process was well‐organized. Also, 83% and 93% agreed or strongly agreed that CARE accelerated their study recruitment and improved the representation of AANHPI participants in their study, respectively. Lastly, 96% of users agreed or strongly agreed they would submit a referral again for future research. Open‐ended responses identified several study‐level characteristics (e.g., in‐person requirement, stringent eligibility criteria) of the referred studies as challenges to successful recruitment from the registry. Collectively, these studies have produced 12 peer‐reviewed publications and 32 abstracts at professional conferences.

**Conclusion:**

Users overall reported positive experiences with the referral process and indicated that CARE can aid recruitment and diversify study samples. CARE provides a promising recruitment source to support studies seeking to include AANHPI participants.

